# Effect of Fermentation on Cyanide and Ethyl Carbamate Contents in Cassava Flour and Evaluation of Their Mass Balance during Lab-Scale Continuous Distillation

**DOI:** 10.3390/foods10051089

**Published:** 2021-05-14

**Authors:** Yan Qin, Beibei Duan, Jung-Ah Shin, Hee-Jin So, Eun-Sik Hong, Hee-Gon Jeong, Jeung-Hee Lee, Ki-Teak Lee

**Affiliations:** 1 Department of Food Science and Technology, Chungnam National University, 99 Daehak-ro, Yuseong-gu, Daejeon 34134, Korea; sdfqy@outlook.com (Y.Q.); just@cnu.ac.kr (B.D.); waigtd@o.cnu.ac.kr (H.-J.S.); hes9730@o.cnu.ac.kr (E.-S.H.); jeonghg@cnu.ac.kr (H.-G.J.); 2 Department of Food Processing and Distribution, Gangneung-Wonju National University, 7 Jukheon-gil, Gangneung, Gangwon-Do 25457, Korea; jashin@gwnu.ac.kr; 3 Department of Food and Nutrition, Daegu University, 201 Daegudae-ro, Gyeonsan-si, Gyeongsangbukdo 38453, Korea; jeunghlee@daegu.ac.kr

**Keywords:** cassava flour, cyanide, ethyl carbamate, distillation, copper chips, mass balance

## Abstract

When cassava is used for the production of distilled spirits through fermentation and distillation, toxic hydrogen cyanide (*HCN*) is released from linamarin and carcinogenic ethyl carbamate is produced. Herein, cyanide and ethyl carbamate contents were monitored during the fermentation and lab-scale continuous distillation processes. Thereafter, mass balance and the influence of copper chips were evaluated. Results showed that 81.5% of cyanide was removed after fermentation. Use of copper chips completely prevented the migration of cyanide into the distilled spirits, while 88.3% of cyanide migrated from the fermented liquid in the absence of copper chips. Formation of ethyl carbamate was significantly promoted during distillation. Most of the produced ethyl carbamate (73.2%) was transferred into the distilled spirits in the absence of copper chips, only 9.6% of the ethyl carbamate was transferred when copper chips were used. Thus, copper chips effectively prevented the migration of cyanide and ethyl carbamate into the distilled spirts during continuous distillation.

## 1. Introduction

Cassava (*Manihot esculenta Crantz*, *Euphorbiaceae*) is an important root crop that serves as a major food in tropics, such as Africa, Asia, and South America [1]. Cassava is also a promising industrial raw material and, hence, economically important, due to the high starch content (15–33%) in its roots [2]. Generally, cassava tubers can be processed into different products such as chips, gari, flour, tapioca, sago, and abacha [3]. Among them, tapioca, the starch product, is widely used in the food industry. Further, cassava starch is a promising source for the production of ethanol. Briefly, starch is converted into glucose by hydrolysis, which is further converted into ethanol by yeasts such as *Saccharomyces cerevisiae* [4,5].

Consumption of cassava products that are not adequately processed has been found to cause cyanide poisoning [6,7]. The toxicity is attributed to the release of hydrogen cyanide (*HCN*) from cyanogenic glycosides, mainly linamarin (95%) and lotaustralin (5%). In intact roots, compartmentalization of linamarin in cell vacuoles and linamarase in cell walls prevents the enzymatic hydrolysis and release of *HCN* [8]. However, when the tissues are disrupted during cassava processing, linamarin is easily hydrolyzed by linamarase into acetone cyanohydrin, which can spontaneously decompose to acetone and *HCN* [9]. After ingestion, the released *HCN* can exert toxic effects by combining with the ferric ion (Fe^3+^) in cytochrome oxidase, thereby inhibiting the utilization of cellular oxygen [10]. In addition, intake of sub-lethal quantities of cyanide from poorly processed cassava products is known to cause several neurological diseases, including ataxic neuropathy, cretinism, and xerophthalmia [11].

Cyanide content in cassava ranges from 53 to 1300 mg *HCN* equivalents/kg DW in leaves [12] and from 10 to 500 mg *HCN* equivalents/kg DW in the root parenchyma [13]. The Food and Agriculture Organization (FAO) and World Health Organization (WHO) have recommended that the *HCN* content in edible cassava flour should not exceed 10 mg/kg [14]. Therefore, to effectively reduce the cyanide content in cassava flesh, processes such as grating and crushing should be carried out before consumption, during which *HCN* gas rapidly releases from linamarin and finally escapes into the air [15].

Ethyl carbamate is a naturally occurring compound in most of the fermented foods and alcoholic beverages [16], such as wine (1–18 µg/L) and scotch whisky (19–90 µg/L). However, a previous study has demonstrated that ethyl carbamate could induce an increase in the incidence of malignant tumors in liver, lung, and harderian glands [17]. Thus, some countries have regulated the ethyl carbamate content in several foods and alcoholic beverages. For example, in Canada, it is 150 ppb for distilled spirits, 30 ppb for wines, and 400 ppb for fruit brandies; in France, it is 150 ppb for distilled spirits and 1000 ppb for fruit branches; in Germany, it is 800 ppb for fruit brandies; and in Korea, it is 30 ppb for wines [18,19]. During fermentation and distillation, the formation of ethyl carbamate in various alcoholic beverages is influenced by several factors, such as precursors (e.g., cyanide, urea, citrulline and carbamyl phosphate, ethanol, etc.), temperature, light [20], microorganisms [21], presence of copper [22], and storage after distillation [23]. Among them, the amount of *HCN* and the use of copper during distillation seem important for the generation of ethyl carbamate, especially when producing distilled spirits from cassava. Previous studies have shown that the migration of *HCN* into the distilled spirits was inhibited by the binding with copper during distillation [24], and the presence of *HCN* was known to influence the generation of ethyl carbamate [20]. However, studies on the degree of migration during continuous distillation based on mass balance are limited. Therefore, in this study, content changes of cyanide and ethyl carbamate in cassava flour during fermentation processes were monitored. Thereafter, the mass balance of cyanide and ethyl carbamate was evaluated after lab-scale continuous distillation by measuring their contents in the fermented liquid, distilled spirits, and distillers’ stillage. In addition, the influence of copper chips on the migration of *HCN* and ethyl carbamate into the distilled spirits was also investigated.

## 2. Materials and Methods

### 2.1. Materials and Chemicals

Cassava flour, liquefying enzyme (Termamyl 2×, 240 Kilo Units Novo alpha-amylase/g of specific activity, Novozymes Korea Inc., Seoul, Korea), β-amylase solution (Saczyme GO 2×, 1,725 Novo Amyloglucosidase Unit/g of specific activity, Novozymes Korea Inc., Seoul, Korea), coenzyme (GU-210, 3,600 Saccharogenic power/g of specific activity, Korea Fermentation Co., Ltd., Seoul, Korea), and Jenico instant yeast (Jenico Foods Co., Ltd., Soeul, Korea) were provided by the Korea Alcohol and Liquor Industry Association (KALIA). Potassium cyanide (KCN) was purchased from Oriental Chemical Industries (Osaka, Japan). Chloramine T, acetic acid and sodium hydroxide (NaOH) were purchased from Daejung Chemicals & Metals Co., Ltd. (Siheung, Korea). 3-Methyl-1-phenyl-5-pyrazolone (pyrazolone) was obtained from Samchun Pure Chemicals Co., Ltd. (Pyeongtaek, Korea). Linamarin (α-hydroxyisobutyronitrile β-d-glucopyranoside), ethanol (≥99.5%), isonicotinic acid and ethylenediaminetetraacetic acid disodium salt dihydrate (EDTA) were purchased from Sigma-Aldrich Korea, Ltd. (Yongin, Korea). Other solvents and reagents used were of analytical grade.

### 2.2. Ethanol Fermentation from Cassava Flour

**Figure 1:**
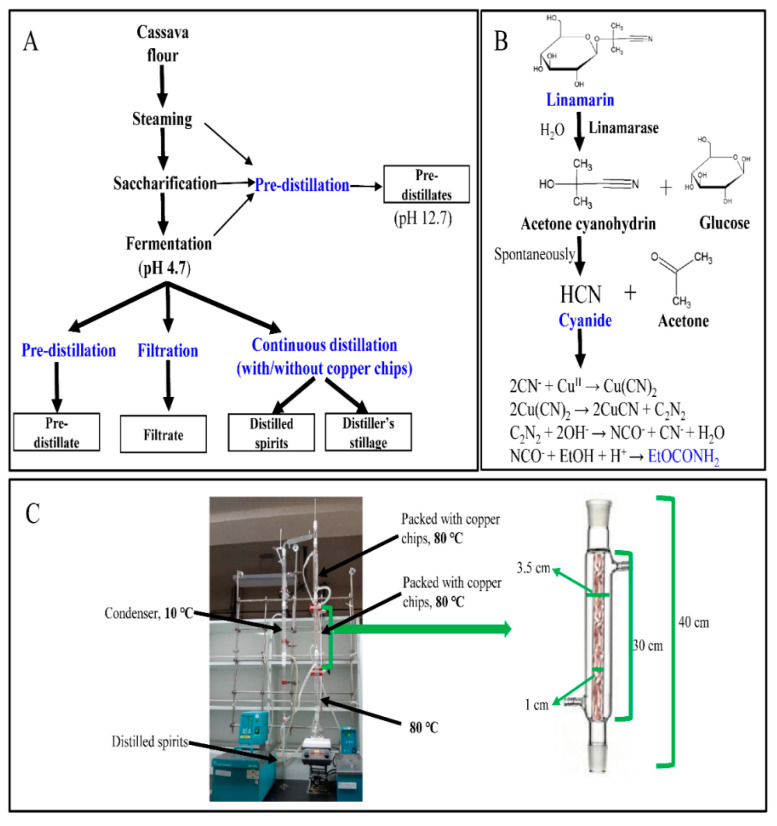
Experimental scheme of ethanol fermentation and continuous distillation (**A**). Hydrolysis process of linamarin and formation of ethyl carbamate from *HCN* catalyzed by copper (II) ions (**B**) [9,25]. Lab-scale continuous distillation apparatus (**C**).

Ethanol fermentation from cassava flour included steaming, saccharification and fermentation processes (Figure 1A), which was performed as follows: cassava flour (10 g) was added into 500 mL round flask, respectively, to prepare total 6 samples. Then, 27 mL of distilled water, 4.625 µL of the liquefying enzyme solution and 0.37 mg of ammonium sulphate were added to each flask. The steaming process was initiated by incubating the samples in an autoclave at 85 °C for 20 min, followed by incubation at 95 °C for 1 h. After that, the autoclave was cooled down to 70 °C and two flasks were taken for duplicate cyanide determination. Thereafter, 37 µL of β-amylase solution and 3.7 mg of the coenzyme were added to the rest 4 flasks for saccharification, which were incubated at 70 °C for 1 h. After incubation, all flasks were cooled down to 33 °C, and two of them were measured for cyanide content. Finally, 3.7 mL of *Saccharomyces cerevisiae* solution (Jenico Instant yeast 1 g/5 mL, activated for 20 min at 37 °C) was added to the remaining two flasks for fermentation, which was conducted in an incubator at 32–33 °C for 4 days. During the ethanol fermentation processes, all flasks were closed with cork and the cassava samples after each process were stored at 4 °C before conducted with pre-distillation. On the other hand, for further lab-scale continuous distillation, 75 g of cassava flour was added into 500 mL flat-bottom flasks, and the fermentation process was conducted after adding 7.5 times of the enzymes and reagents.

### 2.3. Lab-Scale Continuous Distillation

In order to obtain distilled spirits, temperature in the distillation columns should be infinitely close to the boiling point of ethanol (78.2 °C), but as low as possible to that of water (100 °C). In this experiment, the continuous distillation apparatus (Figure 1C) is composed of three distillation columns. Each column has a height of 40 cm, with an outer diameter of 3.5 cm, and an inner diameter of 1 cm. To study the role of copper chips on the migration of cyanide and ethyl carbamate into the distilled spirits, continuous distillation with and without copper chips in the upper two distillation columns were conducted. Herein, the total copper chips packed in two columns was about 17.2 g. During the distillation process, water and ethanol vapors flowed upwards to the distillation columns (80 °C), where most water vapor was condensed and ethanol vapor continued to flow upwards to the next distillation column. The ethanol vapor was finally condensed in the condenser (10 °C) and collected in a measuring cylinder. A thermometer was installed on the top of the distillation column to monitor the top temperature to ensure steady temperature during the distillation process. Moreover, the temperature of the thermometer can also be considered as a sign of the end of distillation, when no vapors reached the top column and the temperature would quickly drop.

To obtain the sufficient distilled spirits, 75 g of cassava flour was fermented and distilled using the continuous distillation apparatus with or without copper chips. The continuous distillation with copper chips in the distillation columns was conducted as follows: a few drops of antifoam were added to the fermented liquid and quickly connected to the distillation apparatus, which was initiated by heating the flask on a hot plate. After approximately 2 h distillation, the volume of distilled spirits was recorded and about 33–34.7 mL of distilled spirits was obtained. The distillers’ stillage was collected after cooling down and filtered twice through a Whatman No. 4 filter paper to obtain the filtrate (158–165 mL). Duplicate distillation was conducted. After that, the copper chips were taken out from the distillation columns, which were washed by deionized water. Thereafter, distillation was conducted without the use of copper chips with the conditions mentioned above. After distillation, all the distilled spirits and filtrates obtained were stored at –20 °C before analysis.

### 2.4. Pre-Distillation for Cyanide Measurement

Pre-distillation prior to colorimetric measurements of cyanide was performed [26]. The principle of pre-distillation was that cyanide can be converted to volatile *HCN* under acidic condition, and further migrated to the pre-distillate by heating. Briefly, cassava flour (10 g) was weighed in a 500 mL round flask, and deionized water (250 mL) was added. The flask was capped and stirred for 2 h to hydrolyze the cyanogenic glycosides. The cassava samples after steaming, saccharification, and fermentation were added with additional deionized water (250 mL). The pre-distillation was performed as follows: each prepared sample was adjusted to a neutral pH, and a few drops of phenolphthalein solution (0.5% in ethanol) were added. Then, ammonium sulfamate solution (10% in water, 1 mL), concentrated phosphoric acid solution (85%, 10 mL), and EDTA NaOH solution (10% in 0.1 M NaOH solution, 10 mL) were added to each flask and maintained for 5 min. After that, each flask was connected to the pre-distillation apparatus and boiled by a heating mantle. Pre-distillate was collected at a rate of 2–3 mL/min in a measuring cylinder (containing 20 mL of 0.5 M NaOH solution), until 90 mL was obtained. Then, the heating mantle was removed, and small amount of deionized water was used to wash the condenser and combined into the pre-distillate. The pre-distillate was finally filled up to 100 mL and stored at –20 °C before analysis.

### 2.5. Cyanide Determination by Colorimetric Methods

Cyanide contents in the pre-distillates of cassava flour and samples (i.e., after steaming, saccharification, and fermentation) were measured by two colorimetric methods. The principle of these methods was that CN^−^ could react with chloramine T and isonicotinic acid-pyrazolone (or pyridine-pyrazolone solution) to form a blue dye at the pH of 7.0. The I-P (isonicotinic acid-pyrazolone) method [27] was conducted using isonicotinic acid-pyrazolone solution (1.5 g of isonicotinic acid dissolved in 24 mL of 0.5 M NaOH solution and filled up to 100 mL with deionized water + 0.25 g pyrazolone dissolved in 20 mL of absolute ethanol). The cyanide standard stock solution (1000 ppm of CN^−^) was prepared by dissolving 2.51 g of KCN in 1 L of deionized water, which was further diluted to 1 ppm as the standard working solution. After that, the cyanide standard solutions were prepared by adding 0, 0.3, 0.6, 0.9, 1.2, 1.5, and 3 mL of the standard working solution into 25 mL colorimetric tubes and filled up to 10 mL by deionized water, while 10 mL of the pre-distillates were added to the tubes. After that, cyanide standard solutions and pre-distillates were added with NaOH solution (0.25 M, 1 mL) and a few drops of 0.5% phenolphthalein solution, and further adjusted by acetic acid solution (0.04%) until the red color started to fade. After adding phosphate buffer solution (0.5 M, 5 mL, pH 7.0) and incubating at 37 °C for 10 min, 0.25 mL of chloramine T solution (1%) and 2 mL of isonicotinic acid-pyrazolone solution were added. Finally, standards and samples to be analysed were filled up to 25 mL with deionized water and incubated at 37 °C for 40 min. The corresponding concentrations of standard solutions were 0, 0.012, 0.024, 0.036, 0.048, 0.060, and 0.120 ppm. All the standard solutions were prepared duplicated. The absorbance was measured by a UV–VIS spectrophotometer (UV-1700, Shimadzu, Kyoto, Japan) at the wavelength of 638 nm.

P-P colorimetric method [26] was performed using a pyridine-pyrazolone solution [0.25 g 3-methyl-1-phenyl-5-pyrazolone solution in 100 mL of deionized water + 0.02 g 0.1% bis (3-methyl-1-phenyl-5-pyrazolone) + 20 mL pyridine]. Briefly, the pre-distillate (20 mL) with a few drops of 0.5% phenolphthalein solution was adjusted by acetic acid solution (1%) until the red color started to fade. Then, 10 mL of phosphate buffer solution (0.5 M, pH 6.8) and 0.25 mL of chloramine T solution (1%) were added. After maintaining at room temperature for 5 min, 15 mL of pyridine-pyrazolone solution was added to the flasks and filled up to 50 mL by deionized water, followed by incubation (25 °C, 30 min). The absorbance was measured at a wavelength of 620 nm. Standard solutions were prepared by diluting to 0.1, 0.2, 0.5, and 1 ppm from the 1000 ppm stock solution, and each solutions were measured for two times. In case of I-P and P-P methods, the pre-distillate samples after fermentation were treated with ethanol evaporation before measurement.

### 2.6. Effect of Ethanol on the Measurement of Cyanide

Cyanide standard solution (1 ppm of CN^−^) was prepared using 95.5% ethanol (Sigma-Aldrich Korea, Ltd., Yongin, Korea). Different volumes of cyanide standard solution were taken, inducing the spiking concentrations of 0.06, 0.15, and 0.3 ppm, respectively. The cyanide standard solutions were then prepared with or without ethanol evaporation as follows: each standard solution was first mixed with NaOH solution (5 mL, 0.05 M) in a 50 mL beaker and maintained for 10 min. The beaker was then heated on a hot plate until 1 mL was remained. The sample was then added into a tube and the beaker was washed with 0.05 M NaOH solution for two times and finally filled up to 10 mL. Thereafter, cyanide content was measured by I-P method and the recovery rates were obtained.

### 2.7. Cyanide Determination by Ion Chromatography

Ion chromatography (IC) was also conducted for cyanide determination. Before injecting to IC, different sample preparations were performed according to their characteristic and purpose of experiment. The filtrates were obtained from the fermented liquid and distillers’ stillage, and prepared by filtering through a Whatman No. 4 filter paper. The pre-distillates were obtained from cassava flour, fermented liquid, and each sample after steaming, saccharification, and fermentation. In addition, for the distilled spirits, ethanol was evaporated before injection as described previously. Dionex Thermo Scientific ICS-5000 chromatographic system (Sunnyvale, CA, USA) with a pulsed amperometric detector was used. Separation was achieved using an IonPac AS15 column (2 × 250 mm, Thermo Fisher Scientific Korea Ltd., Seoul, Korea) with a guard IonPac AG15 column (2 × 50 mm) at 30 °C. Each sample (25 µL) was injected and eluted isocratically by 60 mM NaOH solution at a flow rate of 0.25 mL/min. Calibration curve (CN^−^ concentration of 10 ppb, 50 ppb, 100 ppb, 500 ppb, 1 ppm, 5 ppm, and 10 ppm) was used for quantification.

### 2.8. Ethyl Carbamate Measurement by GC-MS Method

Ethyl carbamate content was measured by gas chromatography-mass spectrometer (GC-MS). Before injecting to the GC-MS, different sample preparations were performed according to their characteristic and purpose of experiment. The filtrates were obtained from the fermented liquid and distillers’ stillage, and prepared by passing through a Whatman No. 4 filter paper. The pre-distillates were obtained from cassava flour, fermented liquid, and each sample after steaming, saccharification, and fermentation. Distilled spirits were used for analysis without any pre-preparation. After that, each prepared sample (5 g) was precisely weighed, followed by addition of 1 mL of 400 ppm butyl carbamate solution (internal standard) and water until the total weight of 40 g was obtained. The mixtures were loaded onto 50 mL Chem Elut^TM^ extraction columns (Agilent Korea Ltd., Seoul, Korea). After 4 min, the columns were eluted using 80 mL of dichloromethane and the eluent was concentrated to 2–3 mL by a rotary evaporator. The eluent was finally concentrated using a Kuderna-Danish tube concentrator. The GC MS-QP2010 Plus (Shimadzu, Kyoto, Japan) system equipped with a DB-Wax column (30 m × 0.25 mm × 0.25 μm; Agilent Korea Ltd., Seoul, Korea) was used for the analysis of ethyl carbamate. The temperatures of the injector, and transfer line and ion source were maintained at 180, and 230 °C, respectively. The oven temperature was programmed as follows: starting from 40 °C for 0.75 min; raised to 60 °C at a rate of 10 °C/min; raised to 150 °C at a rate of 3 °C/min and hold for 5 min; finally raised to 220 °C at a rate of 20 °C/min and hold for 5 min. Helium was used as the carrier gas at a flow rate of 0.9 mL/min. Selected ion monitoring (SIM) acquisition mode was used with the ions of 62, 74, and 89 *m/z*, respectively, among which *m/z* of 62 was used as the quantitative ion. Quantification of ethyl carbamate was conducted based on external calibration curves: Y = 0.0016X + 0.0318 (X from 50 to 1600 ppb), correlation coefficient = 0.9995; Y = 0.0019X + 0.0003 (X from 3.125 to 50 ppb), correlation coefficient = 0.9947. Herein, X was concentration of ethyl carbamate; Y was the peak area ratio of ethyl carbamate and butyl carbamate.

### 2.9. Mass Balance of Cyanide and Ethyl Carbamate during Continuous Distillation

Alcohol content of the distilled spirits was measured by a portable density/specific gravity meter (DA-130N, Kyoto Electronics, Tokyo, Japan). To evaluate the mass balance of cyanide and ethyl carbamate, their contents in the fermented liquid, distilled spirits, and distillers’ stillage were measured, respectively (Figure 1A). The values in the fermented liquid depending on different sample preparation (i.e., pre-distillation and filtration) were obtained from the filtrates and the pre-distillates, respectively. Consequently, for obtaining the mass balance, the content of cyanide from filtrate and ethyl carbamate from pre-distillate were used when considering their contents in the fermented liquid. Herein, mass balance of cyanide and ethyl carbamate were evaluated using the recovery rate (%, RR) after continuous distillation with and without the use of copper chips. RR (%) = (total content detected from the distilled spirits + total content detected from the distillers’ stillage)/total content detected from the fermented liquid × 100. Migration rate into the distilled spirits (%, DSMR) of cyanide and ethyl carbamate were calculated as follows: DSMR% = total content detected from the distilled spirits/total content detected from the fermented liquid × 100. The residual rate of distillers’ stillage (%, DSRR) = RR-DSMR. Moreover, the presence of cyanide and ethyl carbamate in the copper chips, which were used during the continuous distillation, was studied by soaking in 0.5 M NaOH solution for 2 h, followed by IC and GC-MS analysis after filtration.

### 2.10. Reduction of Cyanide by Copper Chips

Cyanide standard solution (5 ppm of CN^−^, 10 mL) and 1 g of copper chips were added into a 50 mL beaker, followed by soaking for 2 h. After that, cyanide content in the supernatant was measured by I-P method. In addition, cyanide content of standard solution in the absence of copper chips was also measured as a control. The experiments were conducted duplicated, and the recovery rates of cyanide were calculated after addition of copper chips.

### 2.11. Identification of Linamarin by Liquid Chromatography-Mass Spectrometry (LC-MS)

Linamarin in the fermented liquid, distilled spirits and distillers’ stillage were analysed using a LC/MS-2020 system (Shimadzu, Kyoto, Japan). Linamarin was separated by a Synergi^TM^ 4 μm Hydro-RP 80 Å column (150 mm × 2.1 mm, Phenomenex Korea Ltd., Seoul, Korea) maintained at 25 °C. 0.1% formic acid in water (A) and 0.1% formic acid in acetonitrile (B) were used as the elution solvents. The elution was started with 2% B, maintaining for 5 min; increased to 100% B in 5 min and hold for 3 min; finally decreased to 2% B in 2 min and maintained for 45 min. Each sample was injected at 5 µL and eluted at a flow rate of 0.3 mL/min. The mass spectrometry was carried out in SIM mode, with the monitoring ion of *m/z* 265 (M+H_2_O) in positive ion mode. The presence of linamarin in each sample was verified based on the retention time and identical mass compared with an authentic standard.

## 3. Results and Discussion

### 3.1. Reduction of Cyanide during Ethanol Fermentation

In this study, two colorimetric methods and an ion chromatography (IC) method were used to measure the cyanide contents in cassava flour and samples processed with steaming, saccharification, and fermentation. Before measurement, each sample was pre-distilled, during which cyanogenic glycosides in cassava (mainly linamarin) were decomposed into *HCN* under acidic conditions. The released *HCN* (boiling point of 25.6 °C) was then evaporated at high temperatures from the fermented liquid and finally collected in a sodium hydroxide solution in the form of CN^−^. After that, the cyanide (CN^−^) content in the pre-distillate was measured by two colorimetric methods based on the König reaction [28,29]. The reaction initially involves the conversion of CN^−^ to cyanogen chloride by chloramine T. The cyanogen chloride can cleave the pyridine ring (pyridine or isonicotinic acid) to produce a 2-pentenedial derivative, which is further hydrolyzed to glutaconic aldehyde. Glutaconic aldehyde can combine with a primary amine or a compound containing reactive methylene hydrogen atoms to form a blue-purple dye. Herein, pyrazolone acts as the methylene hydrogen in the pyridine-pyrazolone (P-P) method and isonicotinic acid-pyrazolone (I-P) method, and absorbance from the final blue solutions are measured at wavelengths of 620 and 638 nm, respectively. Moreover, the cyanide content was measured using IC method. After pre-distillation, the evaporated *HCN* (pKa = 9.21 at 25 °C) [30] was captured in a sodium hydroxide solution, and consequently presented as CN^−^ in the pre-distillates (pH = 12.7).

To determine the process (steaming, saccharification, and fermentation) that led to a significant decrease in the cyanide content, the mass change of cyanide was expressed as the average value measured using the three analysis methods (I-P, P-P, and IC). In Table 1, cyanide contents in each pre-distillate were measured, resulting in a range of 0.35–0.43 mg (i.e., 3.5–4.3 ppm, RSD = 11.2%), 0.10–0.12 mg (1.01–1.16 ppm, RSD = 7.6%), 0.09–0.09 mg (0.85–0.88 ppm, RSD = 1.8%), and 0.06–0.09 mg (0.64–0.85 ppm, RSD = 14.1%). It was found that the relative standard deviation (RSD) of the total cyanide content in each pre-distillate measured by the three methods varied from 1.8% (after saccharification) to 14.1% (after fermentation), indicating that there was not much difference among the three methods since the expected RSD as a function of analyte concentrations ranged from 11 to 15% at 100 ppb to 1 ppm level [31]. In addition, it was found that 10 g of cassava flour (raw material) contained 0.40 mg (i.e., 40 ppm) of cyanide, which was reduced by 72.6% after steaming. This is because most of the linamarin has been hydrolyzed to *HCN*, which easily evaporated due to its low boiling point after the steaming process. Then, the cyanide content gradually decreased after both saccharification and fermentation and finally reduced by 81.5% with respect to that in the raw material, suggesting that still 18.5% of cyanide was remained in the fermented liquid.

According to the National Standard of the People’s Republic of China (GB 5009.36-2016) [27], the high-boiling point organics (presumably including ethanol) in the distilled spirits should be removed by heating (at 120 °C) before performing the coloring reaction for the measurement of cyanide. However, the effect of ethanol on the determination of cyanide content is not clear. Thus, different volumes of cyanide standard solution in the presence or absence of ethanol were measured by I-P method, resulting in final spiked concentrations of 0.06, 0.15, and 0.3 ppm, respectively. Table 2 shows that the recovery rate (%, RR) of CN^−^ ranged from 92.3% to 98.5% when ethanol was removed, and this was within the admissible range of RR (80–110%) at spiked concentrations of 100 ppb to 10 ppm [31]. However, the RR varied from 132.2% to 147.8% if ethanol was not removed from the samples, giving inaccurate results. This is because when the cyanide content is measured by I-P method, the presence of ethanol in a sample may affect the color development reaction. Therefore, all experiments using chloramine T (Table 1) were performed after removing ethanol by heat. Moreover, ethanol content more than 5% in the matrix has been reported to influence the peak shape of cyanide and induce split peak during the IC measurement [32]. Thus, ethanol removal pretreatment will also be applicable for the subsequent cyanide determination in distilled spirits using IC method.

### 3.2. Total Change in Cyanide Content during the Lab-Scale Continuous Distillation with and without Copper Chips

To obtain distilled spirits with 94.4% *v/v* ethanol, 75 g of cassava flour was fermented and distilled using a lab-scale continuous distillation apparatus. The mass balance of cyanide was evaluated by measuring the total cyanide content (mg) in the fermented liquid, distilled spirts, and distillers’ stillage using the IC method (Table 3). Because copper chips could react with cyanide and promote the production of ethyl carbamate in distilled spirits [24], continuous distillation with copper chips packed in the upper two distillation columns was also conducted to understand the role of copper chips. Before performing the distillation, the copper chips were filled in the columns and left for two weeks at room temperature to induce oxidation.

Pre-distillation was performed to measure the cyanide content in the fermented liquid (from 75 g cassava flour). The cyanide contents were similar when the fermented liquid was either filtered (0.60 mg) or pre-distilled (0.56 mg), respectively (Table 3). These values were approximately 8 times higher than that in the fermented liquid obtained from 10 g cassava flour after pre-distillation (Table 1). Moreover, the similar cyanide content in the pre-distillate and filtrate indicated that most of the linamarin was already hydrolyzed by linamarase. Thus, filtration can replace the pre-distillation step during the measurement of cyanide content in the fermented liquid and distillers’ stillage before the IC analysis.

**Figure 2:**
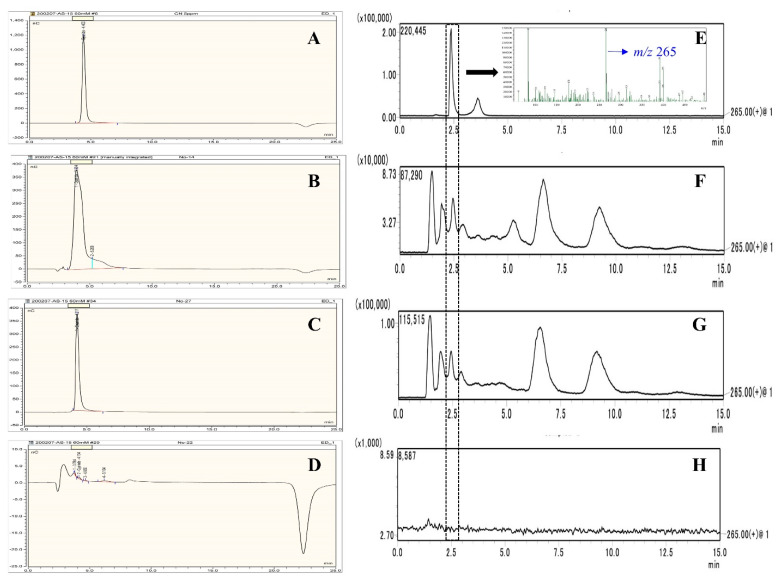
Ion chromatograms of cyanide in standard solution (**A**), pre-distillate containing 20% ethanol (**B**), distilled spirits after ethanol evaporation (**C**), and filtrate of distillers’ stillage (**D**). Ion chromatograms of linamarin in standard (**E**), fermented liquid (**F**), distillers’ stillage (**G**), and distilled spirits (**H**).

Further, distilled spirits and distillers’ stillage were obtained after continuous distillation with or without copper chips and their cyanide contents were examined using the IC method. The cyanide peak cannot be determined due to its co-elution with ethanol (Figure 2B, pre-distillate containing about 20% ethanol). However, when ethanol was removed from the distilled spirits (Figure 2C), the retention time and shape of the cyanide peak were same as that of the cyanide standard (Figure 2A).

Copper chips were found to play an important role in removing cyanide from the distilled spirits (Table 3). Cyanide content in the distilled spirits was 0.53 mg when distillation was conducted in the absence of copper chips. This accounted for more than 88.3% of the total cyanide content (0.60 mg) in the fermented liquid, indicating that most of the *HCN* was transferred into the distilled spirits after continuous distillation. Meanwhile, only a small amount of cyanide (0.03 mg, DSRR = 5.0%, Figure 2D) was detected in the distillers’ stillage, resulting in a RR of 93.3%. On the other hand, when the distillation columns were packed with copper chips, no cyanide was detected in the distilled spirits, and 0.08 mg cyanide (DSRR = 13.3%) was detected in the distillers’ stillage. Therefore, a RR of cyanide with only 13.3% was observed, and from the viewpoint of mass balance, it seems that most of the cyanide was probably present in the copper chips. To confirm this, copper chips were taken out from the columns, and the presence of CN^−^ was examined. It was found that CN^−^ was indeed detected in the copper chips (Table 3), suggesting that the evaporated *HCN* presented in the copper chips.

After fermentation, the *HCN* decomposed from linamarin existed in an unionized form in the fermented liquid owing to its pH of 4.7. During distillation, the volatile *HCN* evaporates with ethanol and water vapor, and comes in contact with the copper chips in the columns. The *HCN* combines with copper [especially copper (II) oxide] and is rapidly oxidized to cyanogen gas (C_2_N_2_), and at the same time, copper (I) cyanide complexes are produced [24]. The copper (I) cyanide complexes are non-volatile insoluble salts, making them difficult to migrate into the distilled spirits; consequently, they are retained in the columns. This finding is also supported by the study by Mackenzie, Clyne, and Macdonald [32], who found the presence of different copper (I) cyanide complexes, such as CuCN, Cu(CN)_2_^−^, Cu_2_(CN)_3_^−^, and Cu_3_(CN)_4_^−^, in grain whisky produced from Coffey still process. However, the consequences might be different if the copper chips are not packed in the distillation columns. The volatized *HCN* would be free to pass through the columns and finally migrate into the distilled spirits. To investigate the effect of copper chips on cyanide, copper chips were added to a 5 ppm CN^−^ standard solution. After 2 h, only 1.91 ppm of cyanide was detected, leading to a RR of 34.1%. However, absence of copper chips led to an acceptable RR of 112.0% (Table 2). Thus, it can be concluded that copper chips can conjugate with cyanide and consequently prevent its migration into the distilled spirits during continuous distillation.

### 3.3. Formation of Ethyl Carbamate during Ethanol Fermentation and Its Migration during Lab-Scale Continuous Distillation with or without Copper Chips

The contents of ethyl carbamate in the pre-distillates were analyzed using GC-MS. These pre-distillates were obtained from 10 g of cassava flour, and samples after steaming, saccharification, and fermentation treatments, which were previously analyzed for cyanide contents. As shown in Table 4, no ethyl carbamate was detected in cassava flour, trace amounts were detected in the pre-distillates after steaming and saccharification, and 0.16 μg was detected after fermentation. However, these values in the pre-distillates might be overestimated, as heat is known to promote the formation of ethyl carbamate [20]. To determine the actual ethyl carbamate content, the fermented liquid (from 75 g cassava flour) was filtered and the content was compared with that of pre-distillates. It was found that 0.34 μg ethyl carbamate was detected in the filtrate, and the content increased significantly to 14.42 μg after pre-distillation. Thus, pre-distillation accompanied by heating would significantly promote the formation of ethyl carbamate. In addition to the effect of heating, the presence of precursors was also important for the formation of ethyl carbamate. As shown in Figure 1B, ethyl carbamate is known to be produced from the reaction of ethanol with cyanate oxidized from *HCN* [24,25]. Thus, the amount of ethyl carbamate in the fermented liquid was slightly higher than those in the steamed and saccharified samples, since ethanol was produced only after fermentation.

The distribution of ethyl carbamate in the distilled spirts and the distillers’ stillage was investigated, and the effect of copper chips on ethyl carbamate formation was also studied. When the fermented liquid was distilled in the presence of copper chips, 1.39 and 14.17 μg of ethyl carbamate were found in the distilled spirits and distillers’ stillage, respectively (Table 4), which were 4−42 times higher compared to those in the fermented liquid (0.34 μg). However, when the fermented liquid was distilled in the absence of copper chips, 10.55 and 2.44 μg of ethyl carbamate were found in the distilled spirits and distillers’ stillage, respectively (Table 4). This was opposite to the trend observed in the presence of copper chips. Consequently, the use of copper chips in the distillation columns significantly reduced the content of ethyl carbamate in the distilled spirits. Moreover, irrespective of the presence or absence of copper chips, the ethyl carbamate contents in the distilled spirits and distillers’ stillage were higher than that in the fermented liquid (0.34 μg), indicating that ethyl carbamate was produced after distillation.

When copper chips were not used during distillation, *HCN* released from the fermented liquid was oxidized to cyanate (OCN^−^), which further reacted with ethanol to produce ethyl carbamate. Therefore, ethyl carbamate is expected to be generated in the distillation column rather than in the heated fermented liquid. In this case, the ethyl carbamate produced in the distillation column would be easily transferred to the distilled spirits by ethanol and water vapor, together with *HCN*. The higher amount of cyanide in the distilled spirits compared to that in the distillers’ stillage in Table 3 also supported the aforementioned phenomenon. On the other hand, 2.44 μg of ethyl carbamate was found in the distillers’ stillage. Ethyl carbamate is highly soluble in water (2 g/mL) and ethanol (1.25 g/mL) [33] and has a boiling point of 185 °C. Therefore, it can be partitioned in the condensed water vapor during the continuous distillation, and some of the produced ethyl carbamate was transferred back to the fermented liquid, thereby allowing it to remain in the distillers’ stillage.

When copper chips were used in the distillation columns, a different phenomenon was observed. During continuous distillation, *HCN* evaporated with ethanol and water vapor and came in contact with copper chips. Copper (especially copper (II) oxide) and *HCN* may combine to form a copper-cyanide complex that can further react with ethanol to produce ethyl carbamate (Figure 1B). The copper chips packed in the distillation columns could lead to effective separation of ethanol from water, owing to which water vapor could easily condense and flow back to the fermented liquid. In the meanwhile, ethanol vapor continued to flow upward, and high-purity ethanol was obtained. Thus, with copper chips, most of the ethyl carbamate produced on the surface of the copper chips was dissolved in the condensed water and flowed back to the distillers’ stillage. Moreover, ethyl carbamate was detected with cyanide in the copper chips after distillation (Table 3 and Table 4). On the other hand, a small amount of ethyl carbamate (1.39 μg) was detected in the distilled spirits despite the use of copper chips and its high boiling point (185 °C). This result may happen due to entrainment. Entrainment refers to the phenomenon in which a liquid is carried upward by the high vapor flow rate during distillation and is finally transferred to the distilled spirits [34]. However, the contents were much lower than those obtained without copper chips. In conclusion, the use of copper chips in the distillation columns could significantly prevent the migration of ethyl carbamate as well as *HCN* into the distilled spirits.

### 3.4. Presence of Linamarin in Samples

Linamarin is widely distributed in cassava cells and is defined as a glycoside of α-hydroxynitriles, mostly D-glucose [35]. The R–CH–CN core of the molecule is derived from an amino acid (i.e., valine) and is further converted to acetone cyanohydrin, which is then glycosylated to form linamarin [36]. The degradation of linamarin begins with the removal of the sugar moiety by the action of a specific β-glycosidase (i.e., linamarase). The resulting cyanohydrins are relatively unstable and can dissociate spontaneously or be catalyzed by hydroxynitrile lyase to produce *HCN*. Table 3 shows that 0.03–0.08 mg of cyanide was detected in the distillers’ stillage after distillation in the absence or presence of copper chips, with DSRR of 5–13.3%. This indicated the possible existence of linamarin in the distillers’ stillage and consequently, in the fermented liquid. It was speculated that linamarin might not be completely hydrolyzed by linamarase. Thus, LC-MS analysis was performed to determine the presence of linamarin in the fermented liquid, distillers’ stillage, and distilled spirits. For this, the fermented liquid and distillers’ stillage were filtered before LC-MS analysis instead of pre-distillation to prevent the hydrolysis of linamarin by acid. Distilled spirits were evaporated to remove ethanol and then re-dissolved in deionized water.

Linamarin was identified by comparing the retention time and mass peak (*m*/*z* of 265) with those of an authentic linamarin standard (Figure 2E). All the samples except distilled spirits (Figure 2H) showed a peak of *m/z* 265 at the same retention time as that of the linamarin standard. Thus, linamarin may present in the fermented liquid and distillers’ stillage but was not in the distilled spirits. The total linamarin content in the fermented liquid and distillers’ stillage was calculated and expressed as *HCN* equivalent as follows:

(1) $$Linamarin\;content\;\left(mg,HCN\;equivelent\right)=\frac{\mathrm{peak}\;\mathrm{height}\;\mathrm{of}\;\mathrm{sample}\times 10\times 27}{\mathrm{peak}\;\mathrm{height}\;\mathrm{of}\;\mathrm{standard}\times 247}\times V,$$

In the formula, 10, 27, and 247 are the concentration of linamarin standard (ppm) and the molecular weight of *HCN* and linamarin (g/mol), respectively; *V* is the volume of sample (L). Although the linamarin contents in fermented liquid and distillers’ stillage were estimated to the greatest extent based on the possible peak height, the values were at most 0.067 and 0.056 mg of *HCN* equivalent, respectively (data not shown). These values were insignificant in terms of the mass balance of *HCN*, indicating that most of the linamarin was hydrolyzed to *HCN* during fermentation.

## 4. Conclusions

In conclusion, cyanide content in cassava flour was found to decrease during ethanol fermentation. Steaming was concluded to be the most effective method, leading to 72.6% reduction in the cyanide content. On the other hand, a small amount of ethyl carbamate was found in the fermented liquid. After continuous distillation in the absence of copper chips, 88.3% of the cyanide and 73.2% of the produced ethyl carbamate were transferred into the distilled spirits. However, when copper chips were packed in the distillation columns, no cyanide was detected in the distilled spirits, and only 13.3% was detected in the distillers’ stillage, suggesting that most of the cyanide combined with copper chips during the distillation. In addition, the DSMR of ethyl carbamate was 9.6%, and most remained in the distillers’ stillage (DSRR = 98.3%). These observations were influenced by several factors such as the physical properties of cyanide and ethyl carbamate (e.g., boiling point, solubility, etc.), type of distillation (i.e., continuous), reaction between copper and cyanide, and distillation conditions. In conclusion, the migration of cyanide and ethyl carbamate into the distilled spirits could be prevented if continuous distillation was conducted in the presence of copper chips.

## Figures and Tables

**Table 1:** Content changes of cyanide (mg) in 10 g cassava flour during the ethanol fermentation processes measured by I-P, P-P and IC method. Unit = mg.

Samples ^1^	I-P ^2^	P-P ^3^	IC ^4^	Average ^5^	RSD ^6^ (%)	Reduction (%)
Cassava flour	0.43 ± 0.07	0.43 ± 0.11	0.35 ± 0.09	0.40 ± 0.05	11.2	0
Steaming	0.10 ± 0.02	0.12 ± 0.02	0.12 ± 0.02	0.11 ± 0.01	7.6	72.6
Saccharification	0.09 ± 0.00	0.09 ± 0.00	0.09 ± 0.01	0.09 ± 0.00	1.8	78.6
Fermentation	0.09 ± 0.00	0.06 ± 0.00	0.08 ± 0.00	0.08 ± 0.01	14.1	81.5

^1^ Samples were the pre-distillates obtained from cassava flour, and cassava after steaming, saccharification and fermentation. ^2^ Isonicotinic acid-pyrazolone method. ^3^ Pyridine-pyrazolone method. ^4^ Ion chromatography method. ^5^ Results were expressed as mean ± SD of the three measurements. ^6^ Relative standard deviation of the three methods. Detailed experimental information is described in Section 2.4 and Section 2.5.

**Table 2:** Effect of copper chips and ethanol evaporation on the recovery rate (%, RR) of cyanide, measured by isonicotinic acid-pyrazolone (I-P) method. Unit = ppm.

	Spiked CN^−^ Concentration	Measured CN^−^ Concentration	RR ^1^ (%)
Absence of copper chips	5.00	5.60 ± 0.00	112.0
Presence of copper chips	5.00	1.91 ± 0.56	34.1
After ethanol evaporation ^2^	0.06	0.06 ± 0.00	98.5
	0.15	0.14 ± 0.01	92.3
	0.30	0.29 ± 0.02	94.9
Before ethanol evaporation ^3^	0.06	0.08 ± 0.04	>100 (132.2)
	0.15	0.22 ± 0.01	>100 (147.8)
	0.30	0.41 ± 0.03	>100 (135.1)

^1^ Recovery rate (%, RR) = measured CN^−^ concentration/spiked CN^−^ concentration × 100. ^2, 3^ KCN dissolved in 95.5% ethanol solution was used. Detailed experimental information is described in Section 2.6 and Section 2.10.

**Table 3:** Total cyanide content (mg) in fermented liquid, distilled spirits and distillers’ stillage measured by ion chromatography. Unit = mg.

	Cyanide Content ^1^	DSMR ^2^ (%)	DSRR ^3^ (%)	RR ^4^ (%)	Copper Chips
Fermented liquid (pre-distillate)	0.56 ± 0.03				
Fermented liquid (filtrate)	0.60 ± 0.02				
Distilled spirits (ethanol evaporation)	0.53 ± 0.04	88.3		93.3	– ^6^
Distillers’ stillage (filtrate)	0.03 ± 0.00		5.0		
Distilled spirits (ethanol evaporation)	ND ^5^	0		13.3	+ ^7^
Distillers’ stillage (filtrate)	0.08 ± 0.01		13.3		
Copper chips	detected				

^1^ Lab-scale continuous distillation was performed on the fermented liquid obtained from 75 g of cassava flour. ^2^ Distilled spirits migration rate (%, DSMR) = cyanide content detected from the distilled spirits/cyanide content detected from the fermented liquid (filtrate) × 100. ^3^ Distillers’ stillage residual rate (%, DSRR) = cyanide content in distillers’ stillage/cyanide content in fermented liquid (filtrate) × 100. ^4^ Recovery rate (%, RR) = DSMR + DSRR. ^5^ Not detected. ^6, 7^ Continuous distillation without or with copper chips. The procedures to obtain the pre-distillates, filtrates, and distilled spirits after ethanol evaporation are described in Section 2.3, Section 2.4, and Section 2.6.

**Table 4:** Formation of ethyl carbamate during the ethanol fermentation and continuous distillation processes. Unit = μg.

Samples	Ethyl Carbamate Content	DSMR ^1^ (%)	DSRR ^2^ (%)	RR ^3^ (%)	Copper Chips
**Ethanol fermentation process ^4^**					
Raw material (pre-distillate)	ND ^5^				
Steaming (pre-distillate)	Trace ^6^				
Saccharification (pre-distillate)	Trace				
Fermentation (pre-distillate)	0.16 ± 0.01				
**Continuous distillation process**					
Fermented liquid (pre-distillate)	14.42 ± 1.41				
Fermented liquid (filtrate)	0.34 ± 0.01				
Distilled spirits	10.55 ± 0.02	73.2		90.1	– ^7^
Distillers’ stillage (filtrate)	2.44 ± 0.07		16.9		
Distilled spirits	1.39 ± 1.76	9.6		107.9	+ ^8^
Distillers’ stillage (filtrate)	14.17 ± 2.05		98.3		
Copper chips	Detected				

^1,2,3^ DSMR, DSRR and RR of ethyl carbamate were calculated based on the content in fermented liquid treated with pre-distillation. ^1^ Distilled spirits migration rate (%, DSMR) = ethyl carbamate content in distilled spirits/ethyl carbamate content in fermented liquid (pre-distillate) × 100. ^2^ Distillers’ stillage residual rate (%, DSRR) = ethyl carbamate content in distillers’ stillage/ethyl carbamate content in fermented liquid (pre-distillate) × 100. ^3^ Recovery rate (%, RR) = DSMR + DSRR. ^4^ Ethanol fermentation was conducted with 10 g of cassava flour, and lab-scale continuous distillation was performed on the fermented liquid obtained from 75 g of cassava flour. ^5^ Not detected. ^6^ Values between LOD and LOQ. ^7,8^ Continuous distillation without or with copper chips. The procedures to obtain pre-distillates, distilled spirits and filtrates are described in Section 2.3, Section 2.4, and Section 2.6.
